# PiggyBac transposon-based polyadenylation-signal trap for genome-wide mutagenesis in mice

**DOI:** 10.1038/srep27788

**Published:** 2016-06-13

**Authors:** Limei Li, Peng Liu, Liangliang Sun, Bin Zhou, Jian Fei

**Affiliations:** 1 Research Center for Translational Medicine, Shanghai East Hospital, Tongji University School of Medicine, Shanghai 200120, China; 2 Department of vascular surgery, Shanghai East Hospital, Tongji University School of Medicine, Shanghai 200120, China; 3 Key Laboratory of Arrhythmias of the Ministry of Education of China, East Hospital, Tongji University School of Medicine, Shanghai, China; 4 Department of Cardiology, East Hospital, Tongji University School of Medicine, Shanghai, China; 5 Department of Endocrinology, Changzheng Hospital, Second Military Medical University, Shanghai, 200003, PR China; 6 Metastasis research institute, Shanghai East Hospital, Tongji University School of Medicine, Shanghai 200120, China; 7 School of Life Science and Technology, Tongji University, Shanghai, China; 8 Shanghai Research Center for Model Organisms, Shanghai, 201203, China

## Abstract

We designed a new type of polyadenylation-signal (PAS) trap vector system in living mice, the piggyBac (PB) (PAS-trapping (EGFP)) gene trapping vector, which takes advantage of the efficient transposition ability of PB and efficient gene trap and insertional mutagenesis of PAS-trapping. The reporter gene of PB(PAS-trapping (EGFP)) is an EGFP gene with its own promoter, but lacking a poly(A) signal. Transgenic mouse lines carrying PB(PAS-trapping (EGFP)) and protamine 1 (Prm1) promoter-driven PB transposase transgenes (Prm1-PBase) were generated by microinjection. Male mice doubly positive for PB(PAS-trapping (EGFP)) and Prm1-PBase were crossed with WT females, generating offspring with various insertion mutations. We found that 44.8% (26/58) of pups were transposon-positive progenies. New transposon integrations comprised 26.9% (7/26) of the transposon-positive progenies. We found that 100% (5/5) of the EGFP fluorescence-positive mice had new trap insertions mediated by a PB transposon in transcriptional units. The direction of the EGFP gene in the vector was consistent with the direction of the endogenous gene reading frame. Furthermore, mice that were EGFP-PCR positive, but EGFP fluorescent negative, did not show successful gene trapping. Thus, the novel PB(PAS-trapping (EGFP)) system is an efficient genome-wide gene-trap mutagenesis in mice.

The Human Genome Project (HGP) was a large-scale project initiated at the end of the 20^th^ century with a focus on gene sequence analysis^1^,^2^,^3^. The HGP had a revolutionary impact on the life sciences and made a significant contribution to the industrialization of modern biomedicine^4^,^5^,^6^. With the sequencing of the genomic DNA of humans and many other organisms, comprehensive studies on gene function, including functional genomics studies, have become an important frontier of life science^7^,^8^,^9^,^10^.

Primary techniques in functional genomics include the use of model biological systems with genomic structure similar to that of humans, ontogeny analysis, studies of the development, structure, and functional characteristics of cells and tissues, and studies of complex biological phenomena and related gene functions at the overall biological level^5^,^8^. Animal models with genetic mutations are commonly used to determine gene function. Numerous methods for generating mutations, including X-rays, chemical mutagenesis, retroviral transfection, and transgenic technology, have been used in gene function research^11^,^12^,^13^,^14^; however, these methods do not produce stable expression, frequently affect multiple genes or lead to chromosomal rearrangements, and cannot provide molecular markers to allow collection of mutation information^12^.

Gene targeting technology to produce a specific gene mutation by homologous recombination in embryonic stem (ES) cells, thus generating genetic knock-out or knock-in animals, is currently one of the most important ways of studying the functions of genes^15^,^16^. Unfortunately, this method is limited by the low probability of homologous recombination and time-consuming animal breeding^17^. Moreover, acquired function-inactivated mutations (null mutations) often produce phenotypes with characteristics different from those of molecular damage associated with diseases. Genome editing technologies that have emerged recently, such as ZFN, Talen, and Crispr-Cas, are widely used to create genetically modified organisms^10^,^18^,^19^,^20^.

Gene trapping technology allows report vectors to be randomly integrated in the genome or label insertion sites, causing insertion inactivation mutations, mainly by utilizing eukaryotic gene promoters or poly(A) signals to gene trap^21^,^22^,^23^. By pre-determined screening criteria, a large number of cells or individual mutations are obtained at very low cost and used to reveal gene expression patterns and gene functions. Gene trapping combined with random mutations, known as random gene targeting, can produce a large number of mutant species in a short time and allows identification of mutant genetic information easily and precisely^24^,^25^,^26^. Due to its simple principles and high efficiency, random gene targeting is a convenient way to generate large-scale gene mutations and has been widely applied in plants, insects, drosophila, and mice^23^,^27^. Gene trapping has been used in studies aimed at discovering the functions of a large number of gene sequences. Selectable markers and reporter genes in gene trapping vectors, including enhancer trapping vectors, promoter trapping vectors, and splice acceptor gene trapping vectors, lack their own promoters and are expressed under the control of the endogenous gene’s promoter. Therefore, the gene trapping strategies mentioned above do not capture genes that are not expressed or expressed at very low levels in target cells. Therefore, new gene trap vector systems with high efficiency will accelerate the pace of studies aimed at determining the functions of genes. The poly(A) trap vector can select a promoter (which is usually constitutively expressed) and capture genes not expressed in the target cells; therefore, it can capture more genes^26^,^28^,^29^.

As part of the mature mRNA, the poly(A) tail is important for exporting the mRNA from the nucleus, inhibiting molecular degradation of mRNA in the cytoplasm, and translation^30^,^31^,^32^. Polyadenylation resulting in a poly(A) tail is essential for production of mature mRNA. The signals required for addition of the poly(A) tail are determined by several cis-elements, including an AAUAAA element and a U-rich upstream sequence element^33^. Mutant poly(A) signals could inhibit mRNA polyadenylation and disrupt transcriptional termination^34^. Therefore, poly(A) signals (PAS) and poly(A) tails are important for proper gene expression^35^. Poly(A) gene trapping is performed by trap vectors without the poly(A) signal of the reporter gene^29^,^36^.

Transposable elements, also known as transposons, are a class of mobile genetic units in the genome. Transposon-mediated mutations in the genomes of plants and animals have been widely reported^37^,^38^. In lower organisms, transposable elements are used to generate transgenic animals and perform insertion mutagenesis^39^,^40^,^41^,^42^,^43^. In comparison, the applications of transposons are quite limited in mammals and other vertebrates because of the lack of an efficient and practical transposition system^44^. Significant effort has been expended to establish an efficient mammal transposon system. In 2005, the piggyBac (PB) DNA transposon was applied in genetic analysis of mice and human cells for the first time, demonstrating a practical and efficient system of mammalian transposable elements and providing a new method for conducting large-scale studies of gene function in mammals^45^. The PB transposon has a length of 2472 bp and contains two terminal domains at both ends, as well as a 2.1-kb reading frame encoding transposase in the middle. PB transposons can be removed from the genome and reinserted at the tetranucleotide TTAA site. The minimal terminal domains, containing a 35-bp 5′-end and 63-bp 3′-end, are important for efficient chromosomal integration by the PB transposon^46^. Previous studies have shown that PB transposons have high transposition activity in several species, including insects, drosophila, and mice^47^. However, transposons such as the P-element transposase from Drosophila have not been widely in mice because of several limitations, including inefficiency, a limited ability to carry gene fragments, and the close proximity of most new transposon insertions to the original site^48^.

In this study, a poly(A) gene trap strategy, known as an expression-independent gene trap, was designed using EGFP as a reporter gene and designated PAS-trapping (EGFP). In order to increase the efficiency of gene trapping and gene targeting, the construct contains an IRER (internal ribosome entry site) cassette to avoid nonsense-mediated mRNA decay (NMD) and an RNA instability element (ARE) to improve splicing efficiency^28^,^49^,^50^,^51^. Then, taking advantage of the efficient transposition ability of PB, we obtained a PB transposon system carrying the PAS-trapping (EGFP) gene trap vector for gene trapping and insertional mutagenesis, which was designated PB(PAS-trapping (EGFP)). PB(PAS-trapping (EGFP)) can be used to produce transgenic mice capable of producing offspring with a wide range of insertional mutations. A primary feature of PB(PAS-trapping (EGFP)) is that PB transposon-mediated integration events within the genome can be screened conveniently with high efficiency almost independent of the insertion site, because the reporter EGFP gene is driven by a constitutively active promoter. The purpose of the PB(PAS-trapping (EGFP)) system is to allow researchers to generate a variety of species with different genetic mutations, which can be used to study gene function and establish disease models, as well as for research and development in aspects of therapeutic drugs. We hope that adoption of this technology will increase the efficiency of forward transposon-based genetic screening and gene targeting in mice.

## Results

### Construction of a poly(A) gene trap (splice donor gene trap) vector with PB transposable sites and corresponding transgenic mice

**Figure 1:**
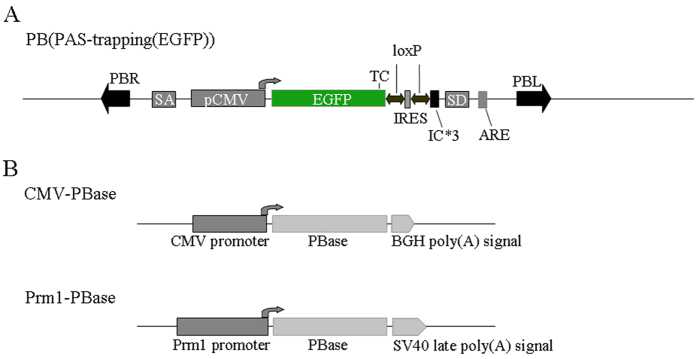
Schematic diagram of PB(PAS-trapping (EGFP)) gene trapping vector and transposase constructs of the piggyBac Transposition System for Mice. (**A**) PB donor constructs, named as PB(PAS-trapping (EGFP)). The reporter gene of PB(PAS-trapping (EGFP)) is an EGFP gene driven by the human CMV promoter. An unstable mRNA signal sequence (ARE) to the 3′ end of EGFP was added. The IRES, three ICs and SD were added between EGFP and ARE. There is the option to remove the IRES using Cre, thereby removing the truncated protein (but not GFP). A SA was added in front of EGFP reporter gene. The PAS-trapping (EGFP) cassette was placed between a pair of PB terminal domains (PBL and PBR, black arrows). (**B**) PB transposase helper constructs. CMV-PBase was constructed by inserting CMV promoters in front of PBase gene, followed by BGH pA. The piggyBac transposase gene (PBase) driven by Prm1 promoters (Prm1-PBase) were followed by SV40 late poly (**A**) signal. (SA: splicing acceptor; CMV promoter: cytomegalovirus immediate early promoter; TC: termination codon; IRES: internal ribosome entry site; IC: initial codon; SD: splicing donor; ARE: an RNA instability element; PBL: PB repeat left termini; PBR: PB repeat right termini; pA: poly (**A**); BGH pA: bovine growth hormone poly (**A**).; Prm1: Protamine 1).

**Figure 2:**
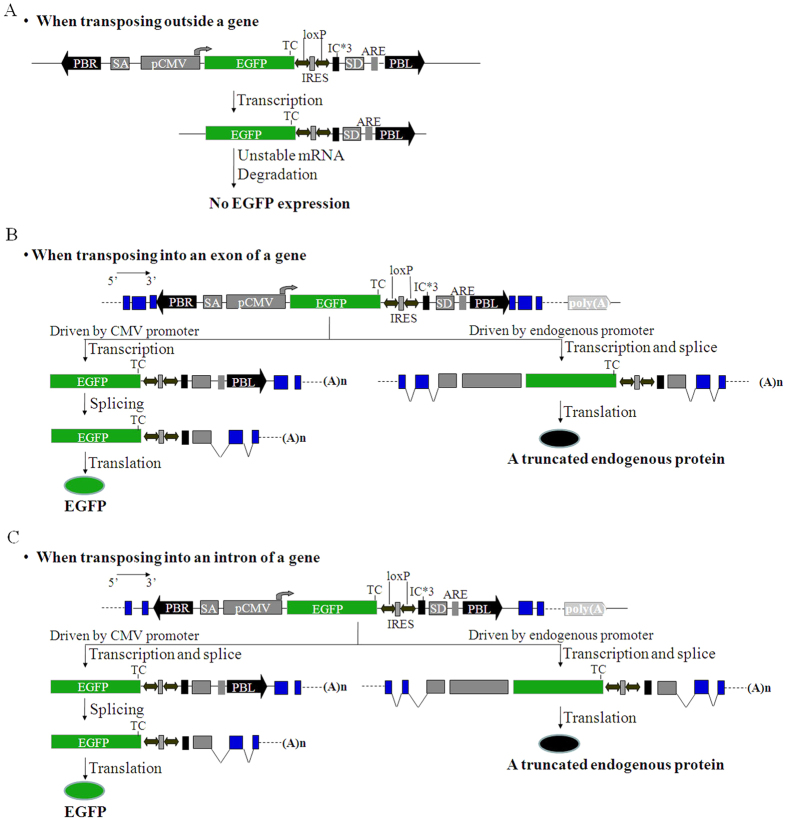
Illustration depicting PB(PAS-trapping (EGFP)) gene trapping strategies and its mechanism of action. (**A**) The lack of a dedicated poly (**A**) signal for the EGFP leads to no EGFP expression after transposition outside a gene. (**B**) Schematic diagram of gene trap and insertional mutation by PB(PAS-trapping (EGFP)) gene trap vector when transposition into an exon of a gene with the same direction of the endogenous gene reading frame. (**C**) Schematic diagram of gene trap and insertional mutation by PB(PAS-trapping (EGFP)) gene trap vector when transposition into an intron of a gene with the same direction of the endogenous gene reading frame.

According to the principle of poly(A) gene trapping, we designed a high efficiency gene trap vector system for gene trapping and gene targeting, the PB-mediated poly(A) gene trap vector (Figs 1 and 2 and Supplementary Table S1). Our gene trapping strategy is based on the theory that a poly(A) sequence signaling for the termination of transcription is essential for the proper expression of a gene^29^. Therefore, the reporter gene (EGFP in our case) should be expressed only after transposition upstream of an endogenous PAS. The reporter gene is an EGFP gene with its own promoter and without a poly(A) signal, which is flanked by a pair of PB terminal domains (PBL and PBR) (Fig. 1A). We chose the human CMV promoter to drive expression of EGFP, which is expressed in most murine cell types (Figs 1A and 2). Thus, genes expressed in the target cell will be captured, but genes not expressed in the target cells will also be captured. The RNA splicing acceptor (SA), a 432-bp sequence between the 2nd intron and 3rd exon of the human B-cell lymphoma 2 gene (Bcl-2)^52^, was connected to the 5′-end of the CMV promoter to avoid losing the gene inactivation derived from vector insertion (Figs 1A and 2C and Figure S1), because mRNA splicing of the endogenous gene spliced it as a part of the intron when PB(PAS-trapping (EGFP)) was inserted into the intron of the gene in mice^26^,^52^,^53^,^54^,^55^. The SA cassette causes insertional mutation when PB(PAS-trapping (EGFP)) is inserted into an intron. An RNA splicing donor (SD) sequence of approximately 174 bp from between the 8th exon and the 8th intron of the murine Hprt gene^49^ was connected to the 3′-end of the EGFP reporter gene (Figs 1A and 2). The SD cassette was important for efficient splicing with the 3′-end of the gene in which PB(PAS-trapping (EGFP)) was inserted.

Conventional poly(A)-trap systems have two primary shortcomings^49^,^51^. One limitation of conventional poly(A)-trap systems is the biased gene trapping that occurs during random integration^49^. Several research groups reported that most screened mutations mediated by conventional poly(A)-trap vectors occurred near the 3′-end of the affected gene^49^,^56^. When the translation termination signal is too far away from the poly(A) signal, mRNA surveillance mechanisms in eukaryotic cells consider the mRNA to be abnormal and degrade it^49^,^55^. Therefore, the reporter gene is usually not translated because of the long distance between the translation termination signal and the captured poly(A) in the case of the insertion of the trap vector into the 5′-region of the gene. Fortunately, an IRES (internal ribosome entry site) sequence and three ICs (initiation codons) in all three reading frames were proven to avoid the mRNA-surveillance mechanism, as described previously^28^,^49^. This IRES sequence, with a LoxP site on both sides and three ICs in all three reading frames, was added between the EGFP report gene and SD sequence in our system (Fig. 1A). Abnormal proteins encoded by IRES mediated by the PB-mediated poly(A) gene trap vector may be harmful in insertion-mutant mice. Therefore, the inserted IRES sequence was deleted using a Cre-mediated LoxP-deletion system. The other primary limitation of conventional poly(A)-trap systems is the production of false-positive clones caused by SD read-through events^51^. Poly(A)-signal trapping vectors obtain false-positive clones by screening with selectable markers or reporter genes if such selectable markers and reporter genes are expressed as a result of transcriptional read-through when they are inserted into a non-gene region or endogenous non-coding 3′-region (Figure S2)^57^,^58^. When vectors with and without an RNA instability element (ARE) were compared, the vector with an ARE showed a reduction of approximately 1.7-fold in the number of trapped G418-resistant colonies in comparison with the vector lacking an ARE^50^,^51^. Therefore, the ARE has the ability to reduce false-positive results^50^. In order to solve this limitation of poly(A) trap vectors, an ARE sequence from human GM-CSF precursor cDNA was added behind the SD sequence (Fig. 1A)^59^. This unstable mRNA signal sequence can lead to instability in mRNA transcribed from EGFP after the trap vector is inserted into a non-gene region or endogenous non-coding 3′-region (Fig. 2A and Figure S2), preventing EGFP protein synthesis^50^,^51^,^57^,^58^.

We constructed transgenic mice with the PB(PAS-trapping (EGFP)) vector. We also produced a PB transposase driven by the CMV promoter (CMV-PBase, Fig. 1B). PB transposons efficiently transpose between vectors and chromosomes via a “cut and paste” mechanism^45^,^60^,^61^,^62^. PB transposons can be removed from vectors and reinserted at tetranucleotide TTAA sites in the genome, preventing concatemer formation during delivery of the vector into chromosomes^61^,^62^. In order to avoid integration of multiple copies of the PB(PAS-trapping (EGFP)) vector in a head-to-tail array at donor sites^63^, we utilized PB transposons for cut-and-paste gene delivery. Before it was microinjected into fertilized mouse oocytes to generate PB(PAS-trapping (EGFP))-positive transgenic mice, the PB(PAS-trapping (EGFP)) vector was mixed with the CMV-PB vector at a mass ratio of 1:2 in the form of a circular plasmid. Genomic DNA was isolated from pup tail samples and used for genotype identification by PCR. We obtained 10 PB(PAS-trapping (EGFP)) single-positive transgenic mice.

### Transposition strategy of PB(PAS-trapping (EGFP)) in male mice

**Figure 3:**
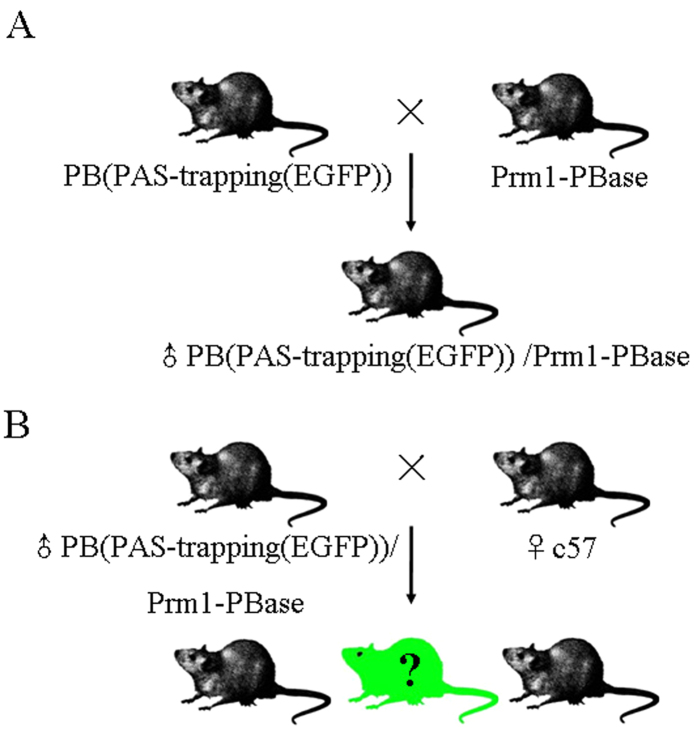
Overview of the breeding scheme used to generate mutant mice in the mouse germline. GFP was used as a marker to monitor transposition events. (**A**) Obtaining the male mice doubly positive for PB(PAS-trapping (EGFP)) and Prm1-PBase. Transgenic mouse lines carrying PB(PAS-trapping (EGFP)) and protamine 1 (Prm1) promoter-driven PB transposase transgenes (Prm1-PBase) were generated by microinjection. The male mice doubly positive for PB(PAS-trapping (EGFP)) and Prm1-PBase were obtained by mating the PB(PAS-trapping (EGFP)) and Prm1-PBase transgenic lines. (**B**) Generating mutant mice in the mouse germline. The mice doubly positive for PB(PAS-trapping (EGFP)) and Prm1-PBase were crossed with WT females to generating offspring with various insertion mutations.

Spermatogonia with various mutations produce sperm with the corresponding mutations via the expression of PB transposase in spermatogonia, allowing corresponding individual mutants to be obtained by subsequent reproduction (Fig. 3). In order to obtain transgenic mice expressing PB transposase in mouse spermatogonia, we selected the mouse prm1 promoter to drive the expression of PB transposase in spermatogonia (Fig. 1B)^64^,^65^ and generated Prm1-PBase mice through transgenic technology. Six founder mice of Prm1-PBase transgenic mice were obtained and crossed with C57 mice. The progenies of Prm1-PBase transgenic founders were screened for PBase expression. Founder mouse no. 5 had the highest PBase expression and was selected for further study. Male mice doubly positive for PB(PAS-trapping (EGFP)) and Prm1-PBase were obtained by mating the PB(PAS-trapping (EGFP)) and Prm1-PBase transgenic mouse lines. With the expression of PB transposase in the male reproductive system, spermatogonia obtain various mutations and produce sperm containing these mutations, so that corresponding mutants are obtained by reproduction (Fig. 3).

### Generation of mice carrying insertion mutations and identification of insertion sites

**Figure 4:**
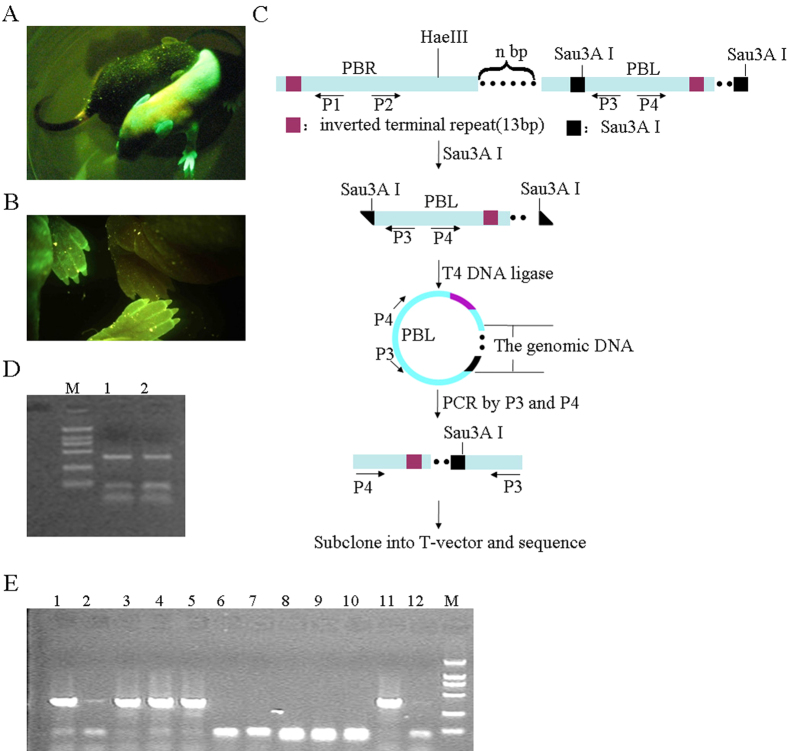
Screening for mutant mice. (**A,B**) Screening for mutant mice performed by GFP expression. Newborn mice were examined by fluorescence microscopy. (**C,D,E**) Molecular analysis of flanking genomic DNA sequences at sites of new transposon insertion. (**C**) Schematic diagram of reverse PCR for identification of insertion sites, after digested with restriction enzyme Sau3A I. (**D**) The PCR products were amplified from ligation products of the SauAI-digested genomic DNA with T4 ligase. (**E**) Clone selection of the positive clones, who had the PCR product (obtained in Fig. 4D) by PCR. After electrophoresis of the PCR products in 1.2% agarose gels, the desired nucleic acid fragments were purified and imported into pMD18-T Simple vector.

LM-PCR was performed to determine the transposition sites of these 10 PB(PAS-trapping (EGFP)) single-positive transgenic mice (Fig. 4). Briefly, tail DNA digested with Sau3AI or HaeIII was ligated, after which transposition sites were amplified by PCR with transposon-specific primers^45^. We found that the PB(PAS-trapping (EGFP)) transposition cassette in PB(PAS-trapping (EGFP)) transgenic founder mouse no. 106 was inserted into the 2nd intron of spastic paraplegia 20 (Spg20) on the 3rd chromosome. However, the EGFP gene had a reading direction opposite that of Spg20; therefore, EGFP protein was not translated (Figure S3A). In order to facilitate subsequent analysis of insertion sites, we selected founder mouse no. 106, which expressed a single copy of the PB(PAS-trapping (EGFP)) transposition cassette at a single insertion site and showed no EGFP fluorescence on the surface, for further study.

**Figure 5:**
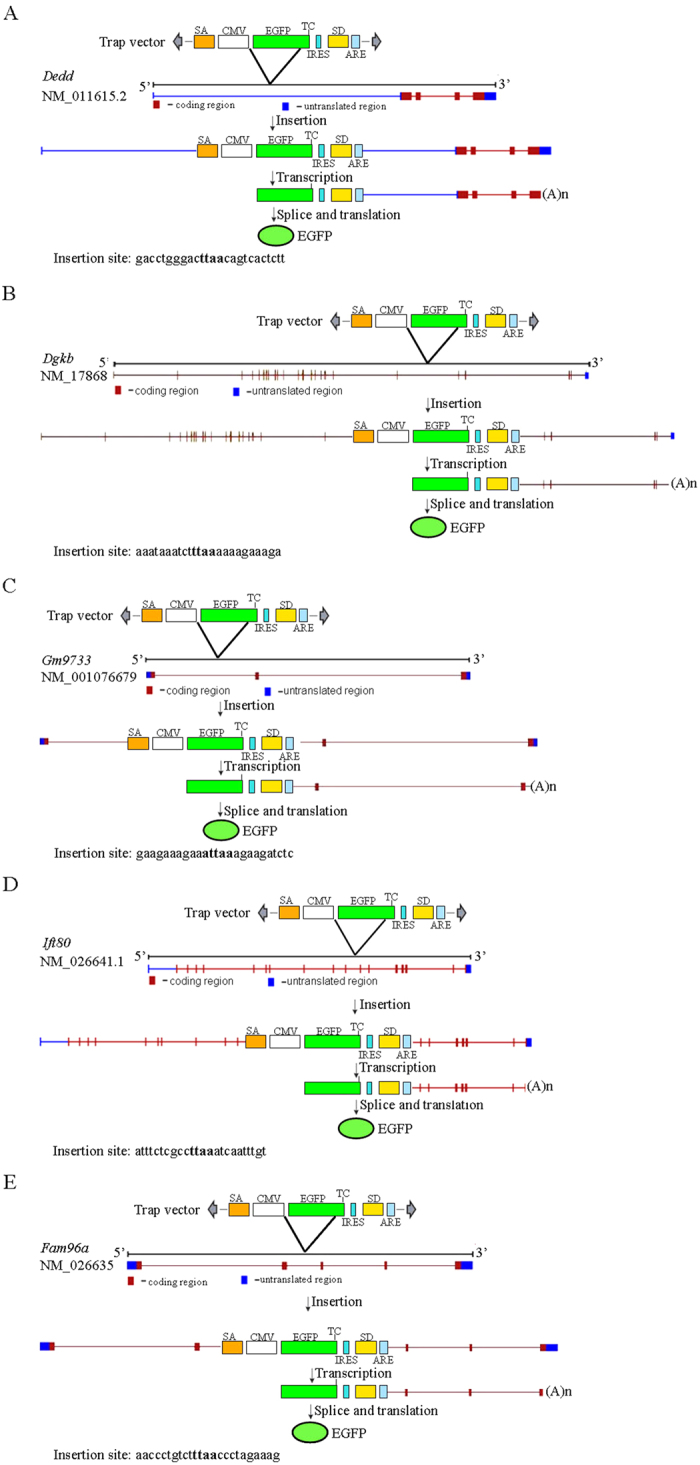
Five independent insertion sites were mapped in EGFP-expressing mice. (**A**) Dedd (inserted in 1th intron) was inserted by PB (PAS-trapping (EGFP)) trap vector. (**B**) Dgkb (inserted in 19th intron) was inserted by PB (PAS-trapping (EGFP)) trap vector. (**C**) Gm9733 (inserted in 1th intron) was inserted by PB (PAS-trapping (EGFP)) trap vector. (**D**) Ift80 (inserted in 11th intron) was inserted by PB (PAS-trapping (EGFP)) trap vector. (**E**) Fam96a (inserted in 2th intron) was inserted by PB (PAS-trapping (EGFP)) trap vector. Dgkb: Diacylglycerol kinase beta, Dedd :death effector domain-containing, Ift80:intraflagellar transport 80 homolog (Chlamydomonas), Gm9733 and Fam96a are putative genes.

PB(PAS-trapping (EGFP)) transgenic founder mouse no. 106 was mated with Prm1-PBase transgenic mice founder mouse no. 5. Two male PB(PAS-trapping (EGFP))/Prm1-PBase transgenic mice were obtained and mated with wild-type C57 mice. Fifty-eight pups from seven litters were screened under a fluorescence microscope and subjected to PCR analysis. One mouse from a litter of eight mice resulting from the pairing of PB(PAS-trapping (EGFP))/Prm1-PBase transgenic mice and wild-type C57 mice emitted significant fluorescence (Fig. 4A,B). Among the eight pups, six PB(PAS-trapping (EGFP)) transgene-positive mice, including the fluorescent subject, were identified by conducting PCR on genomic DNA (Table 1). Transgene insertion sites were identified in the six obtained PB(PAS-trapping (EGFP)) transgene-positive mice using LM-PCR (Fig. 4C,D,F). The PB(PAS-trapping (EGFP)) transposition cassette of the fluorescence-positive mouse was inserted into an intron of the DEDD (death effector domain-containing) gene (Table 2 and Fig. 5A), while the PB(PAS-trapping (EGFP)) transposition cassettes of the remaining five mice were all located in the 2nd intron of Spg20 on the 3rd chromosome (Table 2 and Figure S3A). The reading direction of EGFP in the fluorescence-positive mouse was consistent with that of the DEDD gene. The EGFP reporter gene was expressed only when trapping the poly(A) signal of a gene (Table 2).

We found 26 pups that were trapping vector-positive, of which five pups showed green fluorescence on their body surface. The EGFP reporter gene was expressed only when trapping the poly(A) signal of a gene. Genomic walking analysis confirmed that the EGFP gene was inserted inside an endogenous gene with the same transcription direction in each fluorescent mouse. The five independent insertion sites were mapped in the EGFP-expressing mice: DEDD (inserted in the 1st intron) (Table 2 and Fig. 5A), DGKB (diacylglycerol kinase beta, inserted in the 19st intron) (Table 2 and Fig. 5B), Gm9733 (a putative gene, inserted in the 1st intron) (Table 2 and Fig. 5C), Ift80 (intraflagellar transport protein 80 homolog, inserted in the 11th intron) (Table 2 and Fig. 5D), Fam96a (inserted in the 2nd intron) (Table 2 and Fig. 5E). However, no successful trapping events were identified in the 21 pups that lacked an EGFP signal, of which 19 pups showed the insertion identified in founder mouse no. 106, whereas 2 pups showed a new insertion that was not inserted into any known gene (Figure S3B,C). Our results indicate that EGFP fluorescence on the body surface of newborn mice is a reliable marker for screening endogenous gene-trapped mice.

Among the 58 pups from seven litters mentioned above, we found that 44.8% (26/58) of pups were transposon-positive progenies by conducting PCR on genomic DNA. Identification of insertion sites in transposon-positive mice showed that there were 7 transposon-positive offspring with new transposon integrations and 19 transposon-positive offspring with transposon integrations identical to that of founder mouse no. 106. The transposition capacity (rate of generating a new site or transposition) of the PB(PAS-trapping (EGFP)) system was 26.9% (7/26) (Table 1). About 71.43% (5/7) of the new trap insertions mediated by the PB transposon were in transcriptional units (Table 2). In addition, 100% (5/5) of the EGFP fluorescence-positive mice had new trap insertions mediated by the PB transposon in transcriptional units. Moreover, the direction of the EGFP gene was consistent with the direction of the endogenous gene reading frame.

After comparing the genomic sequences of the seven mice with new insertion sites (Table 1) with sequences from several public databases, we found that the PB transposons of these mice were all inserted into TTAA sites. Four PB transposons were located in chromosome 3, one was located in chromosome 1, one was located in chromosome 10, and one was located in chromosome 12. These results, as well as the fluorescence microscopy observations, show that fluorescence on the body surface of newborn transgenic mice can be observed only when the transposon has been inserted into a gene and the reading frames of EGFP and the endogenous gene are in the same direction (Table 1).

## Discussion

With the rapid accumulation of sequence data from humans, animals, and plants, determination of the biological functions represented by the sequence data has become urgent^3^,^66^. In the field of gene function studies, gene knockout is currently the most widely used research method. However, due to the low throughput of knockout mouse production, the number of types of knockout mice is quite limited^15^,^67^,^68^. Therefore, there is an urgent need for efficient and high-throughput screening methods for discovering unknown genes and analyzing their functions. In the present study, we utilized PB transposable elements and a PAS-trapping (EGFP) gene trapping vector to achieve gene trapping and insertional mutagenesis in mice. Our results demonstrate that PB transposon-mediated poly(A) gene trapping occurred in the reproductive system after mating male PB(PAS-trapping (EGFP))/Prm1-PBase transgenic mice with wild-type female mice (Figs 3 and 4). Using our PB(PAS-trapping (EGFP)) system, numerous pups carrying mutations in different sites were generated, after which captured genes were detected easily under a fluorescence microscope (Fig. 4 and Table 2). There are several advantages to using our method to generate mutant mice: (1) mutant mice can be identified quickly and noninvasively by EGFP fluorescence, (2) mutants can be obtained easily by mating transposon-positive mice with wild-type mice, (3) it requires less labor than producing knockout mice, and (4) it is easy to detect insertion sites by PCR.

The piggyBac transposon system has been successfully used to create random insertion mutant mice^45^,^69^,^70^,^71^,^72^. However, methods of identifying mice with mutant insertions are not well established. In the present study, we designed a poly(A) gene trapping system, in which a target gene is captured using a reporter gene as bait. Insertion of a DNA vector containing a reporter gene into the genome results in inactivation or mutation of the endogenous gene and simultaneously activation or expression of the reporter gene, allowing for identification of insertion mutagenesis^50^,^73^. The reporter gene used in our study was an EGFP gene with a ubiquitously expressed CMV promoter (Fig. 1), which facilitated detection of offspring with insertion mutations by *in vivo* fluorescence microscopy (Figs 3 and 4). Gene trapping strategies can be divided into three main types: enhancer trap, promoter trap, and splice acceptor gene trap. Enhancer trap vectors have not been widely applied in mice because the mutagenesis rate of enhancer traps has not been reported^74^. In contrast, the mutagenesis rate of promoter traps is high, while their exon insertion frequency is very low^75^. Because the insertion occurs in an intron, the gene trap efficiency of splice acceptor gene trap vectors is 50-fold greater than that of promoter traps^73^. However, alternative splicing may cause low-level generation of wild-type transcripts and less efficient allele mutations^76^. The poly(A) trap vector can capture a wider range of genes by using a constitutively expressed promoter to capture genes not expressed in target cells^28^,^29^. In our study, 100% (5/5) of the EGFP fluorescence-positive mice had new trap insertions in transcriptional units (Fig. 5 and Table 2). No gene trap events were identified in mice that were EGFP-PCR-positive, but EGFP fluorescence-negative (Figure S3 and Table 2). Our results demonstrate that the poly(A) signal and poly(A) tail are essential for proper gene expression.

A highly efficient SA was joined to the 5′ end of the CMV promoter to enhance the efficiency of insertion mutation, as described previously^49^,^50^,^52^. This design disrupts endogenous gene function when trapping vectors are inserted into the intron region, as shown in Fig. 2 and Figure S1. In mammalian cells, if the distance between a stop codon and the last exon-exon junction is more than 60 nt, mRNAs with premature stop codons are degraded by nonsense-mediated mRNA decay (NMD)^49^,^52^. In our PB(PAS-trapping (EGFP)) system, an IRES sequence and three ICs were added to avoid the mRNA-surveillance mechanism, as described previously^49^,^50^,^52^. As shown in Fig. 5 and Table 2, we screened five EGFP fluorescent-positive mice, in which the PB(PAS-trapping (EGFP)) trapping vectors were all more than 60 nt from the last exon-exon junction. These results demonstrate that our system allows the use of EGFP to screen mutant mice in which insertion occurred in the first few introns of an endogenous gene (Table 2 and Fig. 5). In order to reduce the number of false-positive results obtained using the fluorescent microscope, an unstable mRNA signal sequence was used to make mRNA transcribed from EGFP unstable when the trapping vector was inserted into a non-gene region^51^,^59^ (Fig. 2 and Figure S2). For example, EGFP fluorescence was not detected in mouse #191 or #503 (Figure S3B,C), in which the PB(PAS-trapping (EGFP)) vectors were not inserted into known genes.

The introduction of the PB(PAS-trapping (EGFP)) system into the reproductive system allows mice to produce offspring with insertional mutations, which can be screened using a high-throughput method. The transposition capacity (rate of generating a new site or transposition) of the progeny of the male PB(PAS-trapping (EGFP))/Prm1-PBase transgenic mice was 26.9% (7/26) (Table 1), which was higher than that of the sleeping beauty (SB) transposition. The SB transposition capacity was about 20%^77^. EGFP expression was not easily detected in some progeny of the SB transposon-transgenic mice^63^,^77^,^78^. EGFP expression may be influenced by DNA methylation or NMD^78^,^79^. However, in our system, five of the seven new trap insertions showed EGFP fluorescence on the body surface.

Our PB(PAS-trapping (EGFP)) system is functional, but has some limitations. For example, the PB(PAS-trapping (EGFP)) system may produce EGFP fluorescence-positive mice in which the PB(PAS-trapping (EGFP)) trapping vector is inserted outside a gene, because of the presence of *cis*-elements for mRNA polyadenylation in the insertion site. However, an unstable mRNA signal sequence was used to reduce the rate of false-positive results^52^,^63^. In addition, the system PB(PAS-trapping (EGFP)) may produce insertion mutagenesis in EGFP fluorescence-negative mice, because the direction of the EGFP gene in the vector may be opposite the direction of the endogenous gene reading frame. Finally, embryonic lethality precluded screening of some mice.

In our system, PB transposon-mediated poly(A) gene trapping occurred in the reproductive system after mating male PB(PAS-trapping (EGFP))/Prm1-PBase transgenic mice with wild-type female mice (Figs 3 and 4). Numerous pups having different PB-insertion sites were generated, after which captured genes were detected easily under a fluorescence microscope (Fig. 4 and Table 2). Moreover, all transposition events occurred exclusively at TTAA sites (Table 2). Consequently, mice with various mutations can be continuously produced, while an embryo bank containing diverse mutants can be established by mating transgenic mice with wild-type mice. This mutant mouse resource will facilitate studies of gene function and allow the establishment of new disease models to support research aimed at producing new therapeutic drugs. The PB(PAS-trapping (EGFP)) transposon system provides a new method for conducting large-scale studies of gene function in mammals. In comparison with traditional methods such as gene knockout and chemical mutagenesis, PB transposon-based poly(A) gene trapping is convenient, fast, and practical. Therefore, the PB(PAS-trapping (EGFP)) system should enable functional genomics research in mice and other higher animals to be conducted more efficiently. In addition, the adoption of this technology will increase the efficiency of forward transposon-based genetic screening in mice.

## Materials and Methods

### Animals

C57BL/6J mice, PB(PAS-trapping (EGFP)) transgenic mice, and PB transposase transgenic mice were obtained from Shanghai Research Center for Model Organisms (Shanghai, China). All animals were housed in a specific-pathogen-free facility with food and water provided *ad libitum*. All animal experiments were approved by the Institutional Animal Care and Use Committee of Shanghai Research Center for Model Organisms and conducted in accordance with government guide-lines for animal care.

### Construction of a poly(A) gene trap (splice donor gene trap) vector with PB transposable sites

Two LoxP sequences were synthesized. The restriction sites for the first LoxP sequence were BglII and SacI, whereas those of the second LoxP sequence were EcoRI and SalI. The two LoxP sequences were inserted into pEGFP-C1 (Clontech Laboratories, Mountain View, CA, USA) to generate pEGFP-C1-LoxP2. IRES was amplified from a pIRES vector (Clontech Laboratories, Mountain View, CA, USA) and cloned into the SacI and EcoRIII sites of pEGFP-C1-LoxP2 to generate pEGFP-C1-LoxP2-IRES. The SD (splicing donor), approximately 174 bp of the sequence between the 8th exon and 8th intron of the hypoxanthine phosphoribosyltransferase 1 (*Hprt*) gene, was amplified from mouse genomic DNA and inserted into the KpnI and ApaI sites of pEGFP-C1-LoxP2-IRES to generate pEGFP-C1-LoxP2-IRES-SD.

The unstable mRNA signal, a 54-bp fragment from the cDNA of human granulocyte-macrophage colony-stimulating factor precursor (GM-CSF precursor), was synthesized and cloned into the XbaI and MluI sites of pEGFP-C1-LoxP2-IRES-SD to generate pEGFP-C1-LoxP2-IRES-SD-in. PB L was amplified from PB3XP3GFPaf and cloned into the XbaI and MluI sites of pEGFP-C1-LoxP2-IRES-SD-in to generate pEGFP-C1-LoxP2-IRES-SD-in-L, as shown in Fig. 1A. PB R was amplified from PB3XP3GFPaf and cloned into the NotI and KpnI sites of pEGFP-C1-LoxP2-IRES-SD-in-L to generate the gene trap vector, PB(PAS-trapping (EGFP)).

The primers used in the construction of the gene trap vector were as follows:

FLoxP1: 5′ gatcttaataacttcgtatagcatacattatacgaagttatgagct 3′

RLoxP1: 5′ cataacttcgtataatgtatgctatacgaagttatttaa 3′

FLoxP2: 5′ aattcataacttcgtatagcatacattatacgaagttatg 3′

RLoxP2: 5′ tcgacataacttcgtataatgtatgctatacgaagttatg 3′

FIRES: 5′ attgagctccctctccctcccccccccta 3′

RIRES: 5′ gctgaattccatccatccatgggtgtgggcaagcttatcatc 3′

FSD: 5′ agtggtaccacttcagggatttgaatgtaag 3′

RSD: 5′ aatgggcccggacgtctgtacttgactacag 3′

FIn: 5′ gatccatcagtaatatttatatatttatatttttaaaatatttatttatttatttatttaagt 3′

RIn: 5′ ctagacttaaataaataaataaataaatattttaaaaatataaatatataaatattactgatg 3′

FpBac L: 5′ ggatctagagtcgacctgcaggcatgcaag 3′

RpBac L: 5′ aagacgcgtgacaatgttcagtgcagagac 3′

FSA: 5′ cgggagctcctgtatctctaagatggctgg 3′

RSA: 5′ gccacgcgttgcatattatttctactgc 3′

FpBac R: 5′ aatggtacccgatgttttgttttgacggac 3′

RpBac R: 5′ aatgcggccgcgatcaaaacgcaaatcgacg 3

### Construction of the PB transposase expression vector

#### Prm1-PB transposase

In order to obtain transgenic mice expressing PB transposase in the spermatogonia, the Prm1 promoter was amplified from C57 mouse genomic DNA^64^,^65^ and cloned into multiple BglII and HindIII cloning sites of pGL3-Basic (Promega, Madison, WI, USA), after which the cDNA of the PB transposase amplified from pMA-Bac^45^ was inserted into the HindIII and XhoI sites to generate pGL3-Prm1-PBase. The correct reading frame was confirmed by sequencing. The mouse Prm1 upstream promoter-driven PB transposase expression vector is shown in Fig. 1B.

The primers used in the construction of the Prm1 upstream promoter-driven PB transposase expression vector were as follows:

FPrm1: 5′ ctaagatcttgttttactagagcccacc 3′

RPrm1: 5′ tcgaagcttcatggtgctggcttggccg 3′

FPBase: 5′ agcaagcttggatgttctttagacgatg 3′

RPBase: 5′ atgctcgagtcagaaacaactttggcac 3′

#### CMV-PB transposase

The PB transposase cDNA was inserted into the HindIII and XhoI sites of pcDNA3.1 to generate CMV promoter-driven PB transposase expression vector CMV-PB.

### Construction of PB(PAS-trapping (EGFP)) and PB transposase transgenic mice

#### Construction of PB(PAS-trapping (EGFP)) transgenic mice

CMV-PB was mixed with PB(PAS-trapping (EGFP)) at a mass ratio of 1:2 in the form of a circular plasmid. The mixed vectors were microinjected into fertilized mouse oocytes, after which the injected eggs were transplanted into the reproductive systems of pseudopregnant mice to produce pregnancy. Genomic DNA was isolated from the tails of newborn pups and used for genotype identification by PCR to identify PB(PAS-trapping (EGFP))-positive transgenic mice.

#### Construction of PB transposase transgenic mice

The Prm1-PBase expression cassette was digested from pGL3-Prm1-PBase with NotI and AlwNI. Positive transgenic mice containing the vector were generated by the microinjection technique described above.

### Construction of a transgenic mouse line doubly positive for PB(PAS-trapping (EGFP)) and Prm1-PBase and generation of mice carrying insertion mutations

PB(PAS-trapping (EGFP)) and Prm1-PBase double-positive transgenic male mice (PB(PAS-trapping (EGFP))/Prm1-PBase) were screened from the offspring of the PB(PAS-trapping (EGFP) and Prm1-PBase single-positive transgenic lines (Fig. 3). The PB(PAS-trapping (EGFP))/Prm1-PBase mice were mated with wild-type female mice to produce offspring with various insertion mutations.

### PCR and sequence analysis

Genomic DNA from the tail of each EGFP-positive mouse was extracted by phenol-chloroform. The concentration of DNA was measured by assessing the absorbance at 260 nm. The DNA were digested with restriction enzyme HaeIII or Sau3AI and stored overnight at 37 °C, followed by inactivation at 68 °C for 45 min and overnight ligation of the digested genomic DNA with T4 ligase at 16 °C (Fig. 4C). The resulting ligation products were amplified by PCR as follows: 94 °C for 5 min 30 s; 35 cycles of 94 °C for 30 s, 58 °C for 30 s, and 70 °C for 3 min; 72 °C for 10 min. After electrophoresis of the PCR products in 1.2% agarose gels, nucleic acid fragments were purified and imported into pMD18-T Simple vector (TaKaRa Bio Inc., Otsu, Shiga, Japan). Finally, after transformation of *Escherichia coli* cells with the pMD18-T Simple vector and clone selection, positive clones were sequenced.

The primers used in identification of insertion sites were as follows:

P1: 5′ cctcgatatacagaccgataaaacacatgc 3′

P2: 5′ agtcagtcagaaacaactttggcacatatc 3′

P3: 5′ cttgaccttgccacagaggactattagagg 3′

P4: 5′ cagtgacacttaccgcattgacaagcacgc 3′

### Observation in mice under a fluorescence microscope

Fluorescence was visualized using an inverted microscope (Nikon Eclipse TE300, Nikon, Tokyo, Japan). After selecting a 510/20-nm bandpass filter for EGFP detection, EGFP-positive mice were identified.

### Bioinformatics analysis

The nucleotide sequences of captured genes were compared with those available from public genome databases, including the Mouse Genome Database at the University of California (UCSC) and Ensembl mouse genome database. In order to determine the specific position of the endogenous gene in which the trapped gene was located, sequence alignment was conducted using the UCSC database. BLAST homology searches were conducted using the non-redundant expressed sequence tag (EST) database of the US National Center of Biotechnology Information (NCBI). EST clone information, including raw data, was obtained from the NCBI Unigene database.

## Additional Information

**How to cite this article**: Li, L. *et al*. PiggyBac transposon-based polyadenylation-signal trap for genome-wide mutagenesis in mice. *Sci. Rep.* **6**, 27788; doi: 10.1038/srep27788 (2016).

## Supplementary Material

Supplementary Information

## Figures and Tables

**Table 1:** Summary of PB transposition in 106# line.

No	EGFP-PCR positive frequency^1^	New insertions frequency^2^
190–197	4/8 = 0.5	1/4 = 0.25
407–413	3/7 = 0.429	1/3 = 0.333
400–406	2/7 = 0.286	1/2 = 0.5
418–426	5/9 = 0.556	1/5 = 0.2
491–506	3/11 = 0.273	1/3 = 0.333
483–490	3/8 = 0.375	1/3 = 0.333
427–434	6/8 = 0.75	1/6 = 0.167
Total	26/58 = 0.448	7/26 = 0.269

^1^EGFP-PCR positive per litter.

^2^New insertion of the EGFP-PCR positive.

**Table 2:** Distribution of transposon insertion sites in the PB(PAS-trapping (EGFP))/Prm1-PBase system.

NO.	Flanking sequences^1^	Chr^2^	Gene Name/GenBank accession number^3^	Intron or Exon^4^	Direction^5^	EGFP fluorescence
106	**TTAA**TTTTTTAAAA	3	Spg20	2^th^ intron	Opposite^6^	Negative^8^
191	**TTAA**AGGGAAAGAA	3	Not inside known gene	/	/	Negative
503	**TTAA**GTAAATCATT	10	Not inside known gene	/	/	Negative
418	**TTAA**AGATTTATTT	12	Dgkb	19^th^ intron	Consistent^7^	Positive^9^
432	**TTAA**CAGTCACTCT	1	Dedd	1^th^ intron	Consistent	Positive
490	**TTAA**TTTCTTTCTT	3	Gm9733	1^th^ intron	Consistent	Positive
506	**TTAA**GGCGAGAAAT	3	Ift80	11^th^ intron	Consistent	Positive
695	**TTAA**AGACAGGGTT	3	Fam96a	2^th^ intron	Consistent	Positive

Spg20: spastic paraplegia 20, Dgkb: Diacylglycerol kinase beta, Dedd: death effector domain-containing, Ift80: intraflagellar transport 80 homolog (Chlamydomonas), Gm9733 and Fam96a: a putative gene.

^1^Nucleotide composition of flanking sequences from PB(PAS-trapping (EGFP)) insertion site.

^2^Distribution of PB(PAS-trapping (EGFP)) insertions in the chromosome.

^3^Gene/GenBank accession number, in which PB(PAS-trapping (EGFP)) was inserted.

^4^Distribution of PB(PAS-trapping (EGFP)) insertions in genes.

^5^The direction of the EGFP gene in the PB(PAS-trapping (EGFP)) vector within the endogenous gene reading frame.

^6^The direction of the EGFP gene in the PB(PAS-trapping (EGFP)) vector was opposite with the direction of the endogenous gene reading frame.

^7^The direction of the EGFP gene in the PB(PAS-trapping (EGFP)) vector was consistent with the direction of the endogenous gene reading frame.

^8^The EGFP fluorescence negative mice.

^9^The EGFP fluorescence positive mice.
