# Supplementary material for: PiggyBac transposon-based polyadenylation-signal trap for genome-wide mutagenesis in mice
Source: Sci Rep. 2016 Jun 13;6:27788. doi: 10.1038/srep27788 (PMC4904408; doi:10.1038/srep27788)
Supplement: Supplementary Information [file srep27788-s1.doc]

**[Supplementary Information](http://www.nature.com/srep/authors/submit.html#supplementary-info)**

**PiggyBac transposon-based polyadenylation-signal trap for genome-wide mutagenesis in mice**

Limei Li^1, 7, 8, #^, Peng Liu^1, 5, #^, Liangliang Sun^6, #^, Bin Zhou^7^, Jian Fei^1, 2, 3, 4^ *

^1^ Research Center for Translational Medicine, Shanghai East Hospital, Tongji University School of Medicine, Shanghai 200120, China;

^2^ Metastasis research institute, Shanghai East Hospital, Tongji University School of Medicine, Shanghai 200120, China;

^3^ School of Life Science and Technology, Tongji University, Shanghai, China;

^4^ Shanghai Research Center for Model Organisms, Shanghai, 201203, China;

^5^ Department of Cardiology, East Hospital, Tongji University School of Medicine, Shanghai, China;

^6^ Department of Endocrinology, Changzheng Hospital, Second Military Medical University, Shanghai, 200003, PR China;

^7^ Department of vascular surgery, Shanghai East Hospital, Tongji University School of Medicine, Shanghai 200120, China;

^8^ Key Laboratory of Arrhythmias of the Ministry of Education of China, East Hospital, Tongji University School of Medicine, Shanghai, China.

^#^ These authors contributed equally to this work.

* Correspondence to: Jian Fei, School of Life Science and Technology, Tongji University, Shanghai 200092, China. , Tel.: +86-21-65980334, Fax: +86-21-65982429, E-mail: jfei@tongji.edu.cn.

| Table S1. Summary of the DNA elements in the PB(PAS-trapping(EGFP)) system | |
| --- | --- |
| Name | Function |
| PBR | PB terminal domains, important for efficient chromosomal integration by PB transposon |
| PBL |
| SA | Splicing acceptor, causing insertional mutation when PB(PAS-trapping(EGFP)) was inserted into an intron |
| pCMV | Driving the expression of EGFP in almost all type of cells |
| EGFP | A reporter gene, suitable for in-vivo screen under microscope |
| IRES | Preventing the mRNA of EGFP degraded by the mRNA-surveillance mechanism, when PB(PAS-trapping(EGFP)) was inserted into the 5' part of a gene |
| IC*3 |
| LoxP | Making IRSE deleted by a Cre-mediated LoxP-deletion system |
| SD | Important for efficient transcriptional splice with the 3' end of a gene, in which PB(PAS-trapping(EGFP)) was inserted |
| mRNA instability | Leading to instability in mRNA transcribed from EGFP after the trap vector is inserted into a non-gene region or endogenous non-coding 3′-region, preventing EGFP protein synthesis |

**
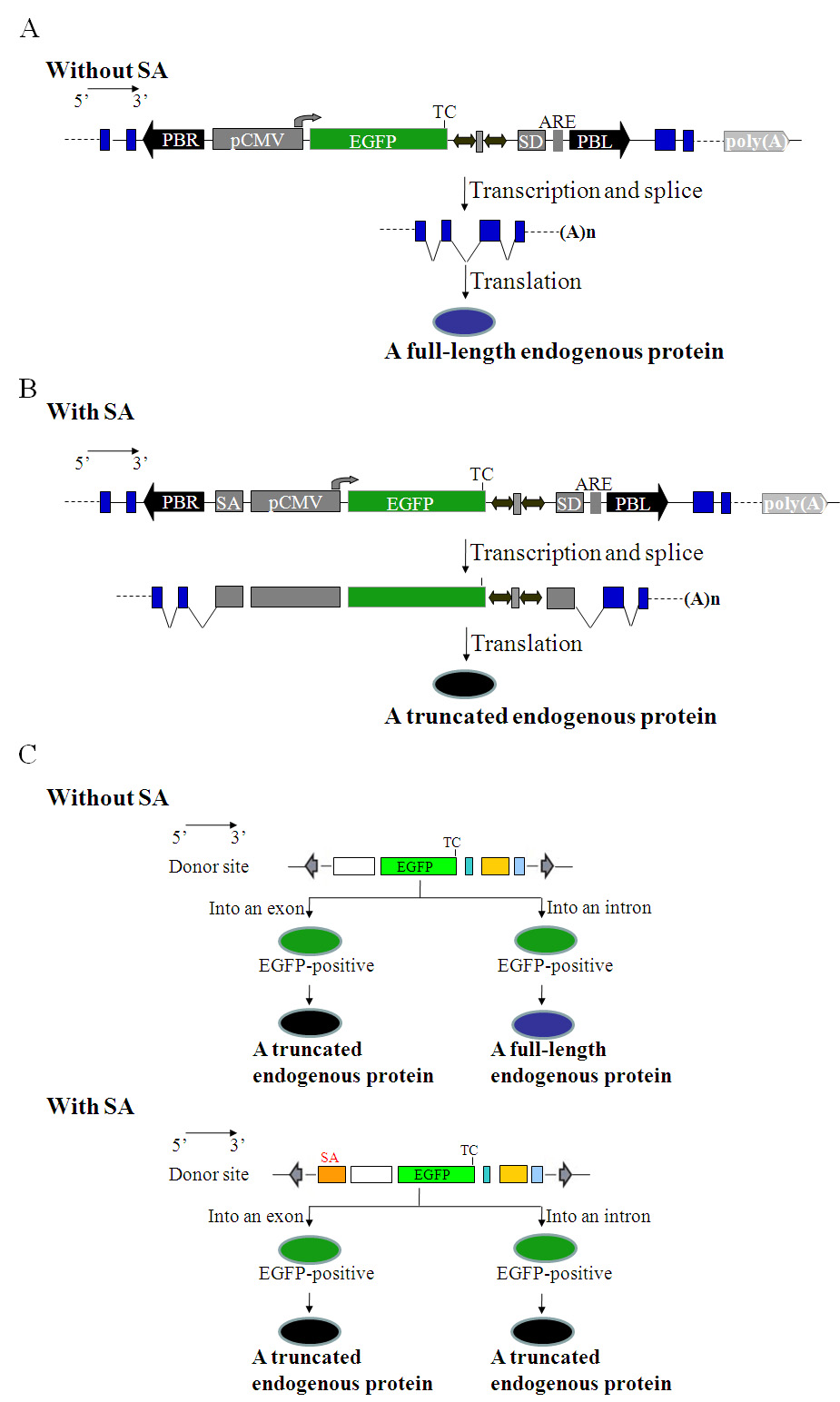
**

**Figure S1. The RNA splicing acceptor (SA) was connected to the 5′-end of the CMV promoter to avoid losing the gene inactivation derived from vector insertion.** (A) mRNA splicing of the endogenous gene spliced the PAS-trapping vector as a part of the intron when PAS-trapping vector without SA was inserted into the intron of the gene in mice. (B) The SA cassette caused insertional mutation when PB(PAS-trapping(EGFP)) was inserted into an intron. (C) The difference between the poly(A) gene trapping vectors with and without SA cassette were compared. (SA: splicing acceptor; CMV promoter: cytomegalovirus immediate early promoter; TC: termination codon; IRES: internal ribosome entry site; IC: initial codon; SD: splicing donor; ARE: an RNA instability element; PBL: PB repeat left termini; PBR: PB repeat right termini; pA: poly(A); BGH pA: bovine growth hormone poly(A).)


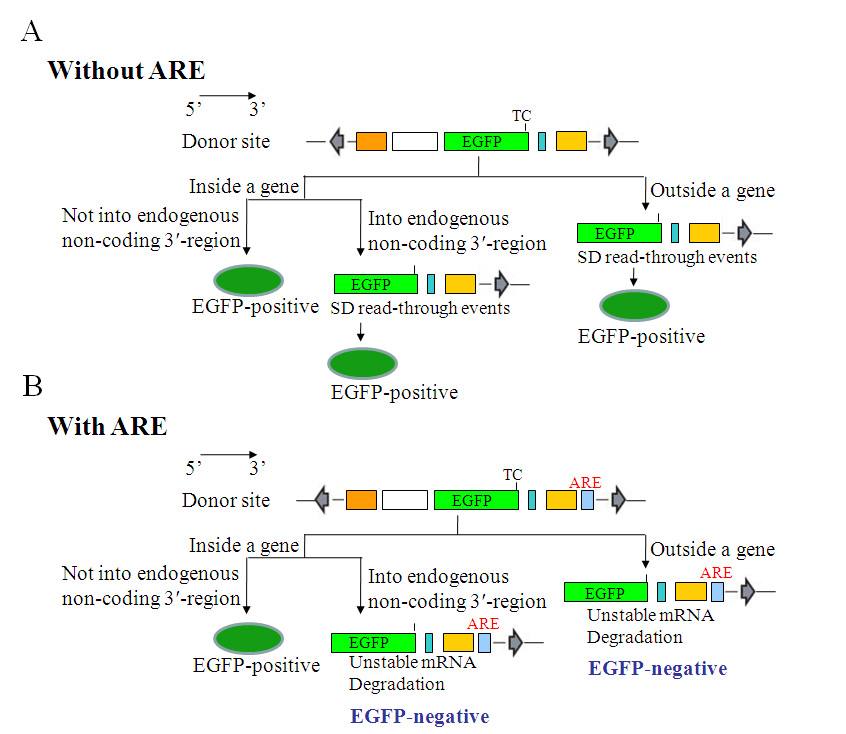


**Figure S2. An ARE sequence has the ability to reduce false-positive results during gene trap by poly(A)-trap systems.** (A) The false-positive clones produced by SD read-through events are obtained by screened with the reporter gene EGFP when the gene trap vector without the ARE element is inserted into a non-gene region or endogenous non-coding 3′-region. (B) The ARE element can lead to instability in mRNA transcribed from EGFP after the trap vector is inserted into a non-gene region or endogenous non-coding 3′-region, preventing EGFP protein synthesis.

**
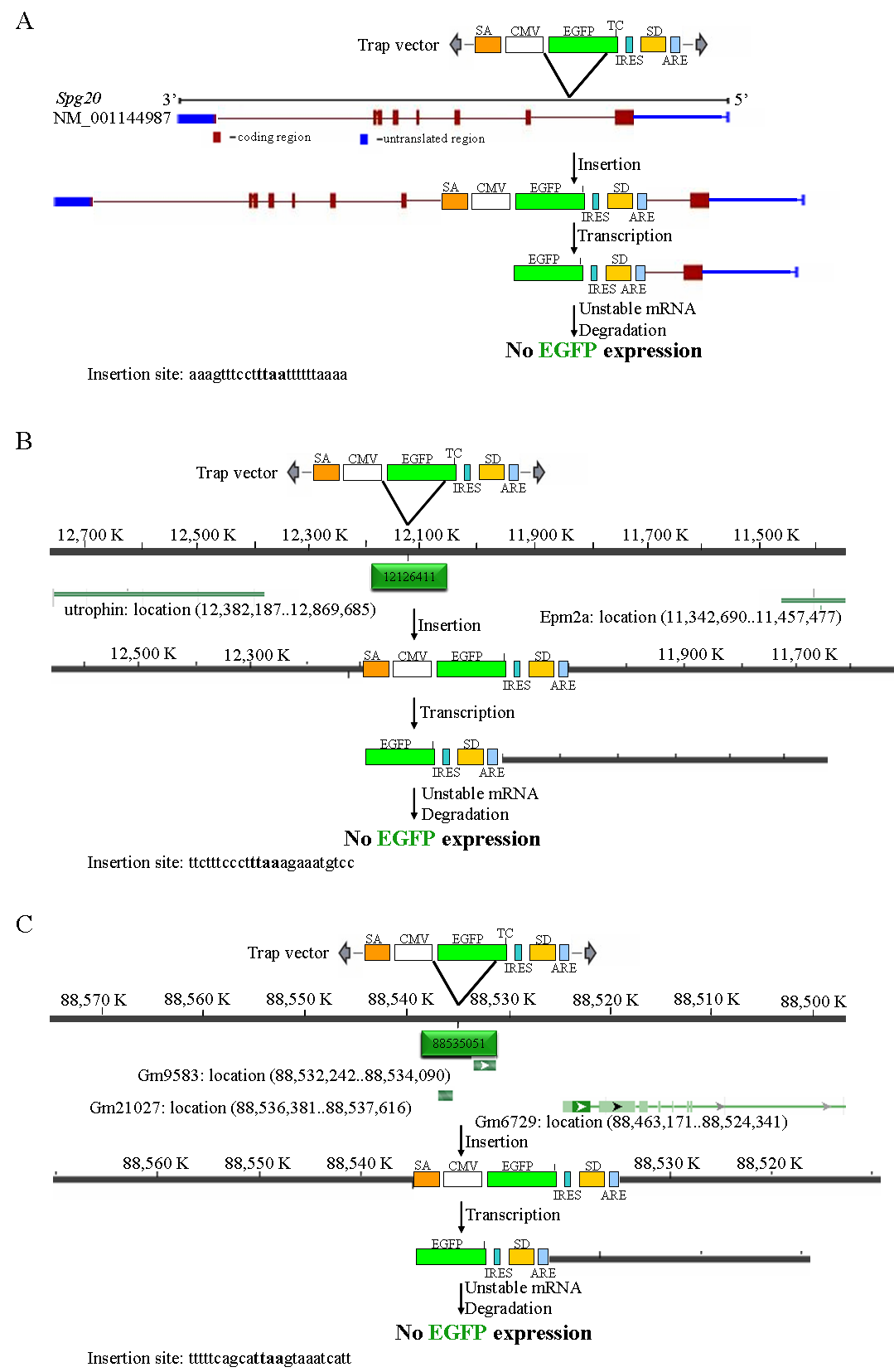
**

**Figure S3. Three independent insertion sites were mapped in no EGFP mice.** (A) The site of #106 mice, which was inserted by PB(PAS-trapping(EGFP)) trap vector. (B) The site of #191 mice, which was inserted by PB(PAS-trapping(EGFP)) trap vector. (C) The site of #503 mice, which was inserted by PB(PAS-trapping(EGFP)) trap vector.
